# Brain activation of the PFC during dual-task walking in stroke patients: A systematic review and meta-analysis of functional near-infrared spectroscopy studies

**DOI:** 10.3389/fnins.2023.1111274

**Published:** 2023-02-16

**Authors:** Qinglei Wang, Wenjun Dai, Sheng Xu, Shizhe Zhu, Youxin Sui, Chaojie Kan, Ying Shen, Yi Zhu, Chuan Guo, Tong Wang

**Affiliations:** ^1^Department of Rehabilitation, The First Affiliated Hospital of Nanjing Medical University, Nanjing, China; ^2^School of Rehabilitation Medicine, Nanjing Medical University, Nanjing, China; ^3^Department of Rehabilitation, Changzhou Dean Hospital, Changzhou, China

**Keywords:** prefrontal cortex (PFC), dual task (DT), walking, functional near-infrared spectroscopy (fNIRS), ischemic stroke, hemorrhagic stroke

## Abstract

**Background:**

Dual-task walking is a good paradigm to measure the walking ability of stroke patients in daily life. It allows for a better observation of brain activation under dual-task walking to assess the impact of the different tasks on the patient when combining with functional near-infrared spectroscopy (fNIRS). This review aims to summarize the cortical change of the prefrontal cortex (PFC) detected in single-task and dual-task walking in stroke patients.

**Methods:**

Six databases (Medline, Embase, PubMed, Web of Science, CINAHL, and Cochrane Library) were systematically searched for relevant studies, from inception to August 2022. Studies that measured the brain activation of single-task and dual-task walking in stroke patients were included. The main outcome of the study was PFC activity measured using fNIRS. In addition, a subgroup analysis was also performed for study characteristics based on HbO to analyze the different effects of disease duration and the type of dual task.

**Results:**

Ten articles were included in the final review, and nine articles were included in the quantitative meta-analysis. The primary analysis showed more significant PFC activation in stroke patients performing dual-task walking than single-task walking (*SMD* = 0.340, *P* = 0.02, *I*^2^ = 7.853%, 95% *CI* = 0.054–0.626). The secondary analysis showed a significant difference in PFC activation when performing dual-task walking and single-task walking in chronic patients (*SMD* = 0.369, *P* = 0.038, *I*^2^ = 13.692%, 95% *CI* = 0.020–0.717), but not in subacute patients (*SMD* = 0.203, *P* = 0.419, *I*^2^ = 0%, 95% *CI* = −0.289–0.696). In addition, performing walking combining serial subtraction (*SMD* = 0.516, *P* < 0.001, *I*^2^ = 0%, 95% *CI* = 0.239–0.794), obstacle crossing (*SMD* = 0.564, *P* = 0.002, *I*^2^ = 0%, 95% *CI* = 0.205–0.903), or a verbal task (*SMD* = 0.654, *P* = 0.009, *I*^2^ = 0%, 95% *CI* = 0.164–1.137) had more PFC activation than single-task walking, while performing the n-back task did not show significant differentiation (*SMD* = 0.203, *P* = 0.419, *I*^2^ = 0%, 95% *CI* = −0.289–0.696).

**Conclusions:**

Different dual-task paradigms produce different levels of dual-task interference in stroke patients with different disease durations, and it is important to choose the matching dual-task type in relation to the walking ability and cognitive ability of the patient, in order to better improve the assessment and training effects.

**Systematic review registration:**

https://www.crd.york.ac.uk/prospero/, identifier: CRD42022356699.

## 1. Introduction

Stroke is the second leading cause of death and disability worldwide (Saini et al., 2021). Many surviving post-stroke patients have residual cognitive impairment and long-term disability that severely affect their normal lives (Delavaran et al., 2017; Young et al., 2020). Studies have shown that more than 50% of patients are unable to walk independently in the community (Blennerhassett et al., 2018). This community ambulation of immersion in daily life requires getting out of the therapeutic environment and interacting with a variety of information (Lord et al., 2004). It goes beyond mere walking and involves motor, sensory and cognitive functions interacting with each other to cope with obstacles and disturbances that may arise during walking to accomplish this multitasking (Caetano et al., 2017). Stroke patients have reduced brain processing capacity due to brain damage, making it difficult for them to multitask (Veldkamp et al., 2021).

Dual-task paradigm is defined as the concurrent performance of two distinct tasks by an individual, and it has been proven that dual-task (DT) walking is a good and valuable paradigm for exploring the walking abilities of stroke patients in daily life (Liu et al., 2018; Bishnoi et al., 2021). As compared to processing one task alone, the performance of one or even both tasks can be degraded when processing two tasks at the same time. This common situation is called dual-task interference (DTI; Tsang et al., 2022). This is manifested in stroke patients by slowed gait speed and increased gait variability (Chatterjee et al., 2019; Hermand et al., 2019). Many theories can be used to explain this occurrence, such as the Capacity theory, Bottleneck theory, or Multiple resource theory (Pashler, 1994; Handy, 2000; Wickens, 2002). However, they have been constructed based on assumed behavioral manifestations and brain capacity. The former has been observed and estimated through many studies on gait parameters. The favorable evidence for the latter needs to be further explored. The study of the neural mechanisms of dual-task is essential for furthering our understanding of how stroke populations cope with DTI.

Brain injury studies have found that patients with prefrontal injury have impaired dual-task execution, but that single-task (ST) processing is not affected (Baddeley et al., 1997). fMRI, PET, and other imaging tools further indicate that specific cortical areas that are associated with dual-task processing include the dorsolateral prefrontal cortex (Kondo et al., 2004; Collette et al., 2005; Low et al., 2009). Elevated metabolic activity in the prefrontal cortex (PFC) has proven that it is strongly associated with increased planning and attention in motor and cognitive tasks, which can verify the changes of brain capacity in theories of DTI (Hamacher et al., 2015). This all points to the value of studying PFC for exploring the effects of dual task in stroke.

Recent neuroimaging techniques have been able to objectively measure the contribution of PFC involvement in human activity (Parris et al., 2019; Udina et al., 2019; Min et al., 2021). Traditional brain imaging techniques such as functional magnetic resonance imaging (fMRI) has a high spatial resolution to detect cerebral blood flow signals. But fMRI has contraindicates of metal implants and they require a more restrictive environment (Mehagnoul-Schipper et al., 2002). EEG has high temporal resolution. But it is less resistant to motion interference and has a lower spatial resolution. It has difficulty in providing a better observation role during a walk. Functional near-infrared spectroscopy (fNIRS) is a new non-invasive optical imaging technique based on the principle of neuro-vascular coupling. When cortical neurons are excited, the oxyhemoglobin (HbO) concentration increases and the deoxyhemoglobin (HbR) concentration decreases, which can indirectly reflects neural activity in the brain (Gramigna et al., 2017). fNIRS has a temporal resolution superior compared to fMRI and can be used in a naturalistic environment. It has better spatial resolution and tolerance to motion artifacts than EEG. fNIRS enables the real-time monitoring of cerebral hemodynamic changes during walking and is not restricted by the freedom to walk (Pinti et al., 2020). So, it can provide a suitable measure of the PFC contribution to walking control (Perrey, 2014). However, fNIRS had limited clinical use due to the lack of anatomic specificity, suboptimal temporal resolution, variable signal-to-noise ratio, and low intra-subject reproducibility for individual analysis (Chen et al., 2020). First, there may be a bias in the anatomical localization of the PFC during the procedure of measuring. Second, it only reflects the overall degree of PFC activation during dual task and cannot detect whether individual neural events occur in the PFC during dual task (Pinti et al., 2020). Third, the hemodynamic response has a delay of 2 s after the occurrence of the event (Jasdzewski et al., 2003). So, it was not temporally synchronized with the occurrence of the event. Fourth, fNIRS has shallow imaging depth. The imaging depth of fNIRS is generally limited to the surface of the cortex in the case of humans (Kim et al., 2017).

Despite the growing body of studies on dual tasks, most of the existing studies performed various types of dual-task paradigms, including motor dual-task, computational dual-task, memory dual-task, and verbal dual-task (Al-Yahya et al., 2016; Hawkins et al., 2018; Hermand et al., 2020). Even for the same pattern, the specific tasks chosen varied across studies. It is not clear whether there are similarities and differences in the degree of PFC activation brought about by these different paradigms in stroke patients. There is a lack of basis for suggestions what dual task should be selected in future clinics. Secondly, with the recovery of the disease duration, the walking activation patterns will be different in different stroke patients (Beyaert et al., 2015). There are no studies directly observing the differences in brain area activation between patients in the subacute and chronic phases while performing dual tasks, which should be explored further. The relevant reviews are mostly qualitative descriptions of the activation of brain regions and the type of tasks (Lim et al., 2021; Veldkamp et al., 2021). The quantitative analysis of the dual task was also about spatiotemopral gait parameters such as walking speed and walking distance, with few fNIRS metrics (Tsang et al., 2022). In addition to that, many studies are currently conducted on dual-task training (Pang et al., 2018; Iqbal et al., 2020; Collett et al., 2021). Brain imaging evidence still to be explored whether it is an appropriate matching of the patient's abilities to the dual-task paradigm for assessment and effective training. The published meta-analysis related to the relationship between PFC and dual task has mainly focused on the elderly and Parkinson's patients, with insufficient inclusion of studies on stroke. It does not reveal well the situation of stroke as a group. We aimed to explore the above questions by analyzing related studies through meta-analysis.

The primary objective of this meta-analysis was to identify the difference of brain activation in PFC between single-task walking and dual-task walking in stroke patients. In addition, the secondary objective was to use subgroup analyses to assess the effects of different dual-task types and disease durations on the differences in activation between stroke patients performing single task and dual task.

## 2. Materials and methods

The results of this systematic review and meta-analysis are reported in accordance with the Preferred Reporting Items for Systematic Reviews and Meta-Analysis (PRISMA) guidelines (Moher et al., 2009). In addition, it was registered on the International Prospective Register of Systematic Reviews (PROSPERO; registration number: CRD42022356699).

### 2.1. Study selection criteria

We used the PICOS framework to formulate the inclusion criteria. Studies that met the following criteria were included in the review: (1) Population: adult participants with stroke. (2) Intervention: Participants in the studies performing dual-task walking (walking whilst performing a cognitive task or another type of motor task). (3) Comparison: The control group was a single walking task. Other tasks were not mixed during the walking. (4) Outcome: Studies used fNIRS to quantify the concentration changes of oxygenated hemoglobin (HbO) in PFC during single-task or dual-task walking. (5) Studies: The study design and the time of publication were not limited. The following types of articles were excluded: (1) conference proceedings, (2) review articles, (3) case reports, (4) retrospective studies, or (5) not written in English.

### 2.2. Search strategy

Keyword searches were performed in Medline, Embase, PubMed, Web of Science, CINAHL, and Cochrane Library from inception to August 2022. The search algorithm included all possible combinations of keywords from the following three groups: (1) stroke; (2) dual task, walking, gait, locomotion, mobility, ambulation, lower limb movement, lower limb motor; and (3) fNIRS, functional near infrared spectroscopy, functional near-infrared spectroscopy, NIRS, near-infrared spectroscopy, near infrared spectroscopy. Keywords and medical subject headings (MeSH) terms were used in the search. Relevant professional journals were searched manually when necessary. The specific search algorithm for each database is provided in Supplementary material.

### 2.3. Data extraction

A standardized data extraction form was used to collect the following methodological and outcome variables from each included study: author(s), year of publication, study design, type of pathology, sample size, participant characteristics (i.e., age, type of stroke, and disease duration), type of dual-task, and fNIRS outcome. Single-task walking was defined as the control group, while dual-task walking was defined as the experimental group. The subacute phase is defined as the range between 7 and 180 days after initial stroke. Chronic stroke is defined as the open-ended time period starting 180 days after initial stroke (Bernhardt et al., 2017). Most data were entered as mean with standard deviation (SD) for both groups at the baseline and when performing the task. When the authors analyzed patients according to gender or functional status categories, we combined the data for patients performing the same task type using the following formula:


(1) $$\begin{array}{l} Combined\;mean=\frac{N1^{*}M1\;+\;N2^{*}M2}{N1\;+\;N2} \end{array}$$

(2) $$\begin{array}{l} Combined\;Standard\;Deviation=\sqrt{\frac{{\left(N1-1\right)}^{*}{S1}^{2}+{\left(N2-1\right)}^{*}{S2}^{2}}{N1+N2-2}\;} \end{array}$$

*N*, the size of sample; *M*, the mean of sample, *S*, the standard deviation of sample.

For the missing data, we tried to contact the authors to obtain the original data. HbO has been reported to be more sensitive to changes in cortical activity associated with walking, while HbR is more susceptible to contamination by factors affecting optical path length and crosstalk than HbO (Leff et al., 2011). For these reasons, HbO was generally chosen as the primary outcome of prefrontal recruitment (Hoshi et al., 2001). Therefore, we only extracted HbO but not HbR to explore the activation of PFC. For randomized controlled trial (RCT) designed studies, only pre-test data were extracted in order to avoid the influence of intervention on PFC (Moon et al., 2016). To avoid the effect of the intervention on the pattern of stroke processing dual task, post-intervention HbO data were not extracted.

### 2.4. Quantitative data synthesis

Meta-analysis was used to determine the pooled effect size of PFC activation comparing single tasks and dual tasks, to measure activation differences between single tasks and dual tasks in stroke patients (ST-DT diff). For each trial in the articles, we used Comprehensive Meta-Analysis (CMA) software version 2.0 (Biostat, Inc., Englewood, NJ, USA) to synthesize an effect size. The overall effect of group comparisons was assessed using the standardized difference in means (SMD) and 95% confidence intervals (CI).


(3) $$\begin{array}{l} SMD=\frac{M1i-M2i}{\sqrt{\frac{\left[{\left(N1i-1\right)}^{*}S{1i}^{2}+{\left(N2i-1\right)}^{*}S{2i}^{2}\right]}{\left(N1i+N2i-2\right)}}} \end{array}$$

The effect sizes were interpreted as 0.2 for a small effect, 0.5 for a moderate effect, and 0.8 or greater for a large effect (Pei and Wu, 2019). We used a random effects model to correct for variable effect sizes due to the heterogeneity in included studies (e.g., characteristics of patients, walking environment, time, or outcome measures). The *I*^2^ index was used to assess the study heterogeneity. In addition, we performed subgroup analyses for study characteristics (disease duration, type of dual-task, or hemispheres) to complete secondary analyses on the impacts of different factors. Publication bias was assessed using a visual inspection of funnel plots. Studies that fell outside the funnel shape had a high risk of bias.

### 2.5. Study quality assessment

The National Institutes of Health (NIH) Research Quality Assessment tool was used to assess the quality of each included study by two reviewers (QL and WJ). The “Quality Assessment Tool for Observational Cohort and Cross-Sectional Studies” was used for cross-sectional studies. The “Quality Assessment of Controlled Intervention Studies” was used for RCT. Each tool consisted of 14 questions. The study quality assessment helped to measure the strength of scientific evidence, but it was not used to determine the inclusion of studies. The GRADEPro (McMaster University, 2020, Ontario, Canada) was used for assessing the quality of the evidence. When two reviewers had a disagreement, a third person (SX) was consulted to resolve it.

## 3. Results

### 3.1. Selection process

**Figure 1:**
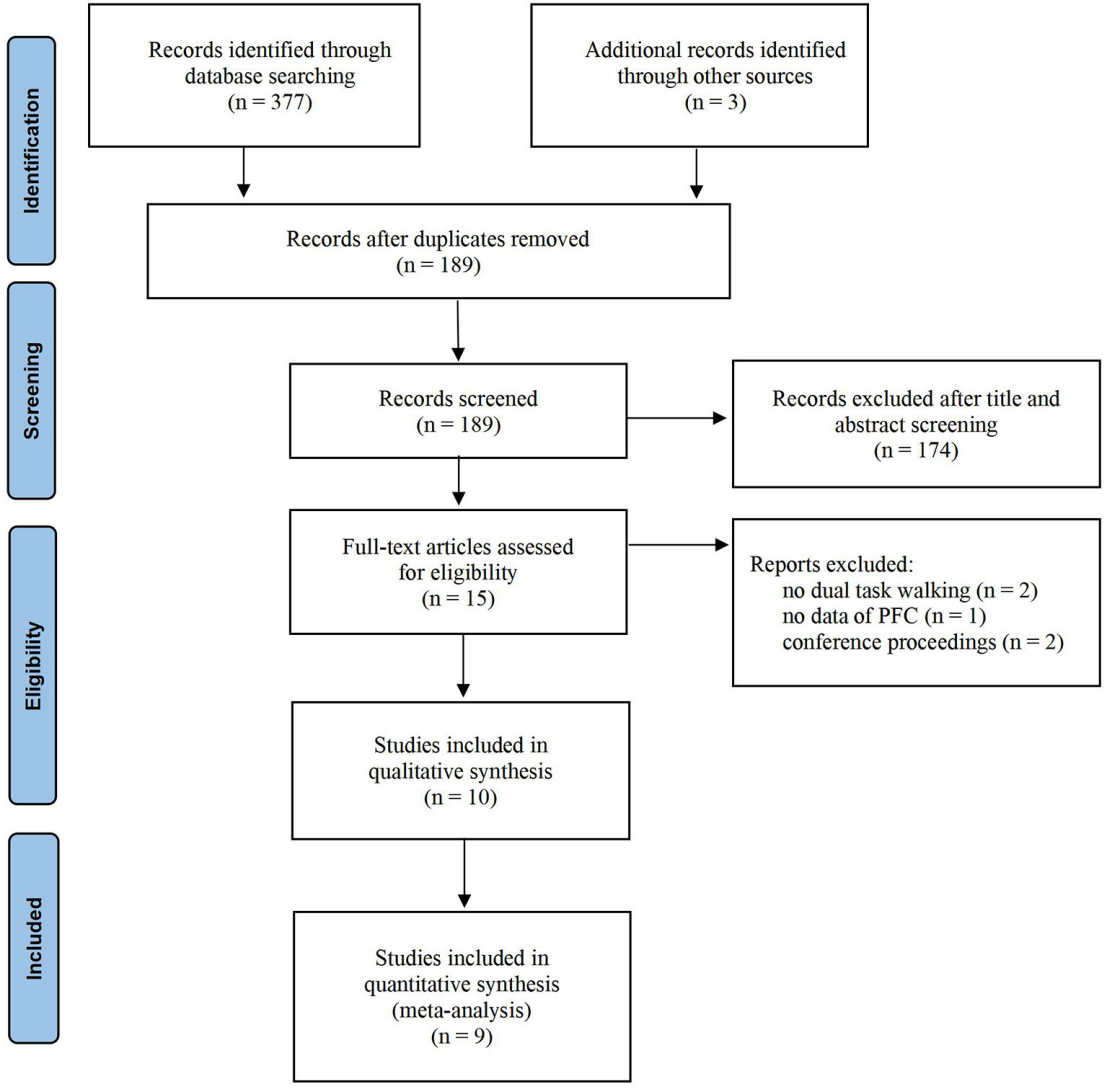
Systematic review, PRISMA flow diagram.

A total of 377 articles were retrieved in the database from six databases, and three articles were identified through other sources. After removing duplicates, 189 eligible records were retrieved. In total, 174 articles were excluded from the title and abstract screening. After full-text reading, five articles were excluded because they did not meet the criteria. Finally, 10 articles were included in the review, and nine articles were included in the meta-quantitative analysis. One paper was excluded from the meta-analysis because it only performed the difference value between HbO and HbR without reporting the HbO data separately. The detailed process is shown in Figure 1.

### 3.2. Basic characteristics of the included studies

Tables 1, 2 summarize the information extracted from the 10 included articles. Eight articles were cross-sectional studies and two articles were RCTs. A total of 224 stroke patients were involved, including 32 patients in the subacute stage and 192 patients in the chronic stage. Five articles adopted the dual-task walking involving serial subtraction. Two articles adopted walking whilst obstacle crossing (Hawkins et al., 2018; Clark et al., 2021). Two articles adopted walking whilst n-back (Hermand et al., 2019, 2020). Two articles adopted walking whilst a verbal task (Hawkins et al., 2018; Lim et al., 2022). Only one paper adopted walking whilst picture-planning or stroop (Collett et al., 2021).

**Table 1:** Study and participant characteristics.

**Study name**	**Study design**	**Participant characteristics**			***N***
		**Age**	**Type of stroke**	**Disease duration**	
Al-Yahya et al. (2016)	Cross-sectional	59.61 ± 15.03	Ischemic; Hemorrhagic	26.5 ± 27.46^a^	19
Hawkins et al. (2018)	Cross-sectional	58.0 ± 9.3	Ischemic; Hemorrhagic	18.3 ± 9.3^a^	24
Liu et al. (2018)	Cross-sectional	51.5	Ischemic; Hemorrhagic	41.5^a^	23
Mori et al. (2018)	Cross-sectional	61.1 ± 9.3	Ischemic; Hemorrhagic	NR	14
Chatterjee et al. (2019)	Cross-sectional	59.6 ± 9.7	Ischemic; Hemorrhagic	19.2 ± 10.4^a^	33
Hermand et al. (2019)	Cross-sectional	71.4 ± 10.1	Ischemic; Hemorrhagic	45.5 ± 34.5^b^	11
Hermand et al. (2020)	Cross-sectional	68.1 ± 9.4	Ischemic; Hemorrhagic	62.92 ± 32.61^b^	21
Clark et al. (2021)	RCT	59.6 ± 9.15	Ischemic; Hemorrhagic	18.00 ± 10.48^a^	38
Collett et al. (2021)	RCT	62 ± 14	Ischemic; Hemorrhagic	51 ± 59^a^	21
Lim et al. (2022)	Cross-sectional	64 ± 7.6	Ischemic; Hemorrhagic	82 ± 67.4^a^	20

^a^Represents the units are months.

^b^Represents the units are days.

RCT, randomized controlled trial; N, the size of sample; NR, not reported.

**Table 2:** Dual-task type and HbO data extraction.

**Study name**	**Type of dual task**	**Representative area**	**ST**		**DT**		**Unit**	***N***
			**Mean**	**SD**	**Mean**	**SD**		
Al-Yahya et al. (2016)	①SS7	Average L and R	0.69	0.96	1.02	1.22	μmol/L	19
Chatterjee et al. (2019)	①SS7	Average L and R	0.26	0.52	0.92	0.98	μmol/L	33
Clark et al. (2021)	①SS7 ②Obstacles	Average L and R	0.34	0.89	0.84 0.8	0.95 1	μmol/L	38 38
Hawkins et al. (2018)	①Verbal ②Obstacles	Average L and R	0.2	0.58	0.68 0.72	0.98 0.59	μmol/L	24 24
Hermand et al. (2019)	①1-back ②2-back	Average L and R	2.42	1.93	2 2.69	2.24 2.22	μmol/L	11 11
Hermand et al. (2020)	②2-back	Affected	NR	NR	NR	NR	μmol/L	21
	②2-back	Un-affected	1.04	1.02	1.49	1.65	μmol/L	21
Collett et al. (2021)	①Stroop ②Planning	Affected	0.67	0.26	0.33 0.42	0.38 0.36	mmol/L	15 15
	①Stroop ②Planning	Unaffected	0.64	0.3	0.49 0.34	0.32 0.39	mmol/L	13 13
Lim et al. (2022)	①Easy verbal ②Hard verbal	Affected	0.14	0.33	0.24 0.25	0.31 0.33	μmol/L	20 20
	①Easy verbal ②Hard verbal	Unaffected	0.06	0.29	0.21 0.18	0.3 0.29	μmol/L	20 20
Mori et al. (2018)	②SS3	Average L and R	−0.3	1.73	−0.073	0.41	AU	14

ST, single-task; DT, dual-task; N, the size of sample; SD, standard deviation; SS7, serial-7 subtraction during walking; Obstacles, walking across obstacles; Stroop, auditory stroop task whilst walking; Planning, picture-planning whilst walking; SS3, serial-3 subtraction during walking; NR, not reported; L, left hemisphere; R, right hemisphere; affected, affected hemisphere; unaffected, unaffected hemisphere.

### 3.3. Meta-analysis

#### 3.3.1. Primary and secondary analyses

##### 3.3.1.1. Activation of PFC: ST vs. DT

**Figure 2:**
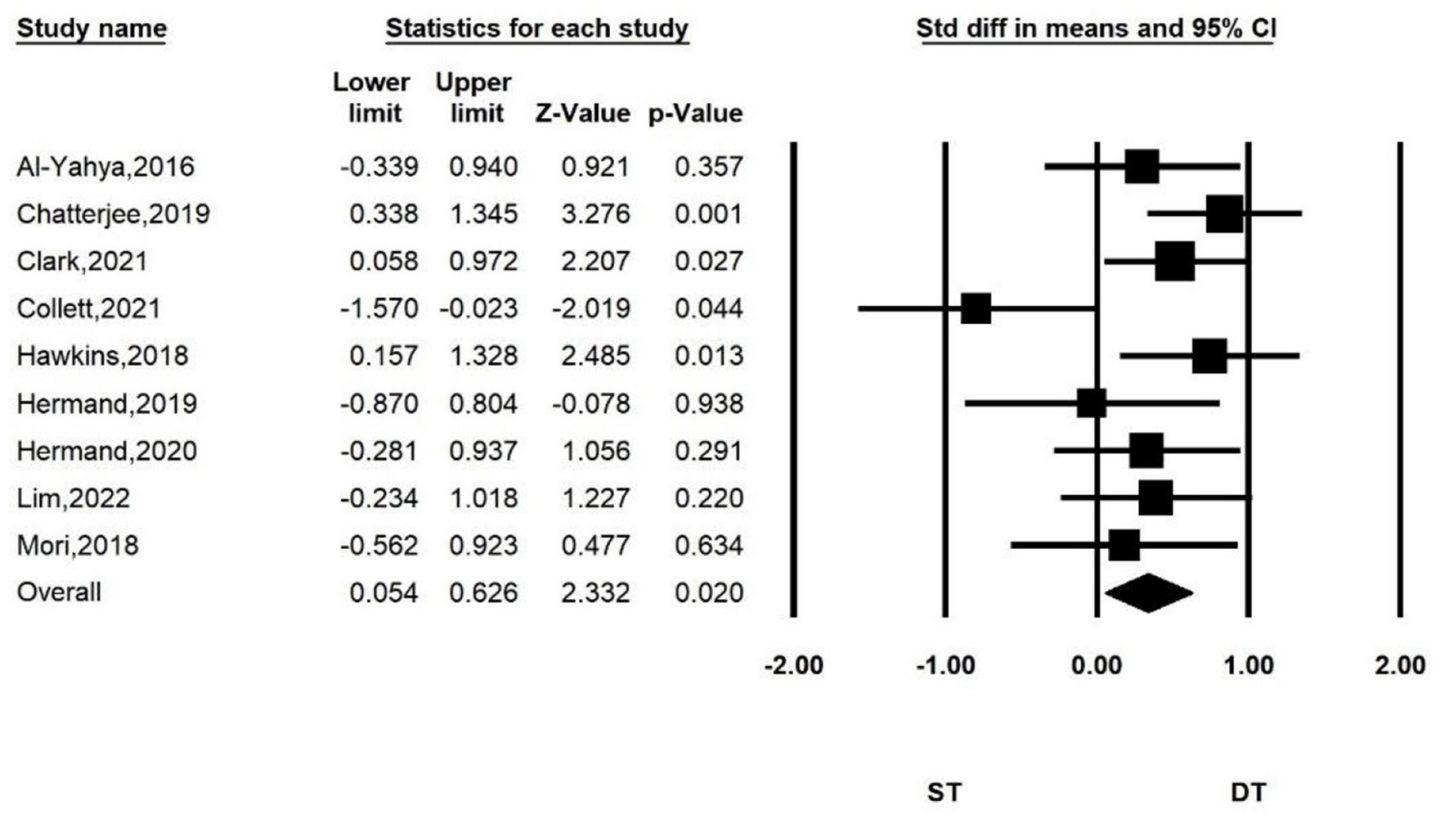
Forest plot for the PFC activation of DT group compared with ST group.

Figure 2 shows the overall meta-analysis of comparing the PFC activation between ST and DT. We found that there was more PFC activation when performing dual-task walking than when performing single-task walking. The total number of studies included in the random effect model was nine. Compared with the ST group, the overall effect size of HbO increase in the DT group was significant (*SMD* = 0.340, *P* = 0.02, *I*^2^ = 7.853%, 95% *CI* = 0.054–0.626).

##### 3.3.1.2. Variations of ST-DT diff: Comparing different disease durations

**Figure 3:**
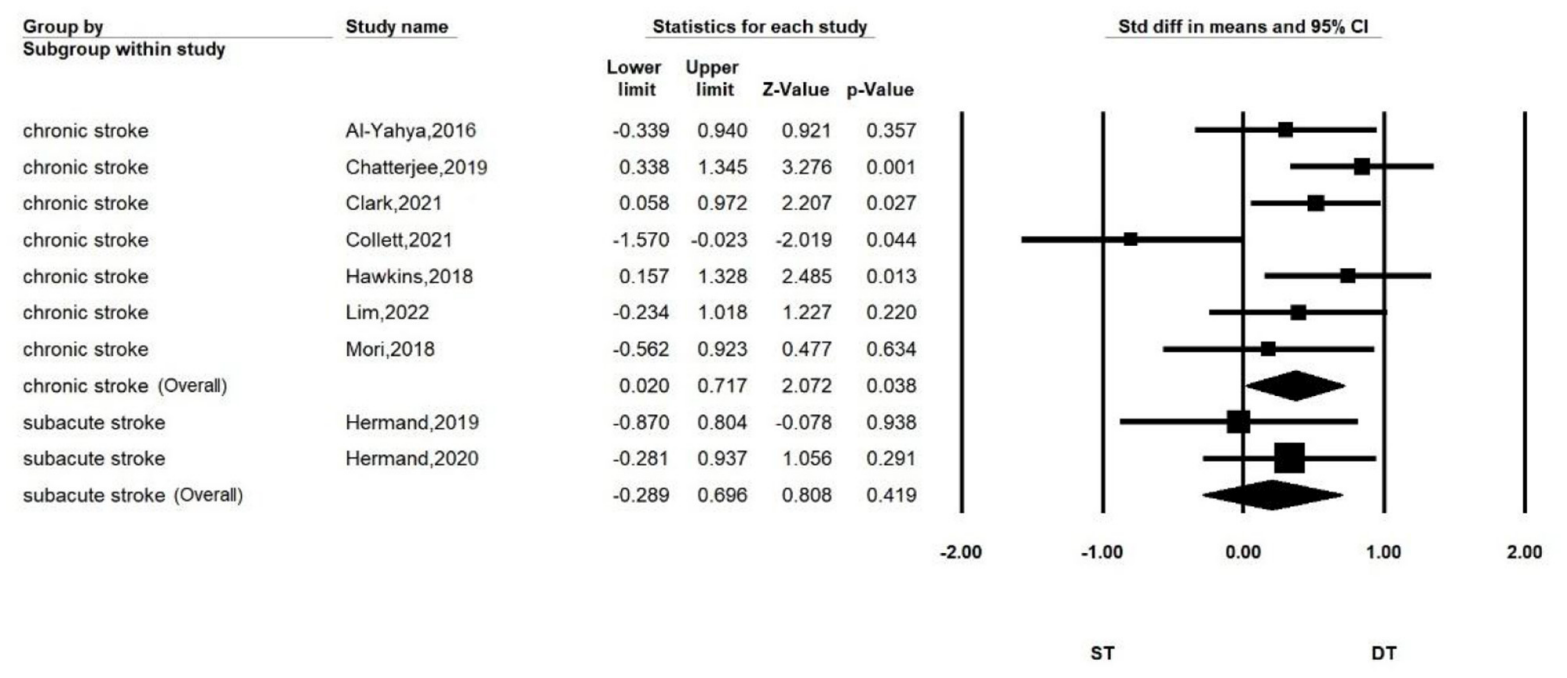
Forest plot for comparing PFC activation of ST and DT in subacute and chronic patients.

Subgroup analyses according to disease duration are shown in Figure 3. The ST-DT diff is significant for stroke patients in the chronic phase (*SMD* = 0.369, *P* = 0.038, *I*^2^ = 13.692%, 95%*CI* = 0.020–0.717), while no significant ST-DT diff was found in patients with subacute stroke (*SMD* = 0.203, *P* = 0.419, *I*^2^ = 0%, 95% *CI* = −0.289–0.696).

##### 3.3.1.3. Variations of ST-DT diff: Comparing different types of dual-task

**Figure 4:**
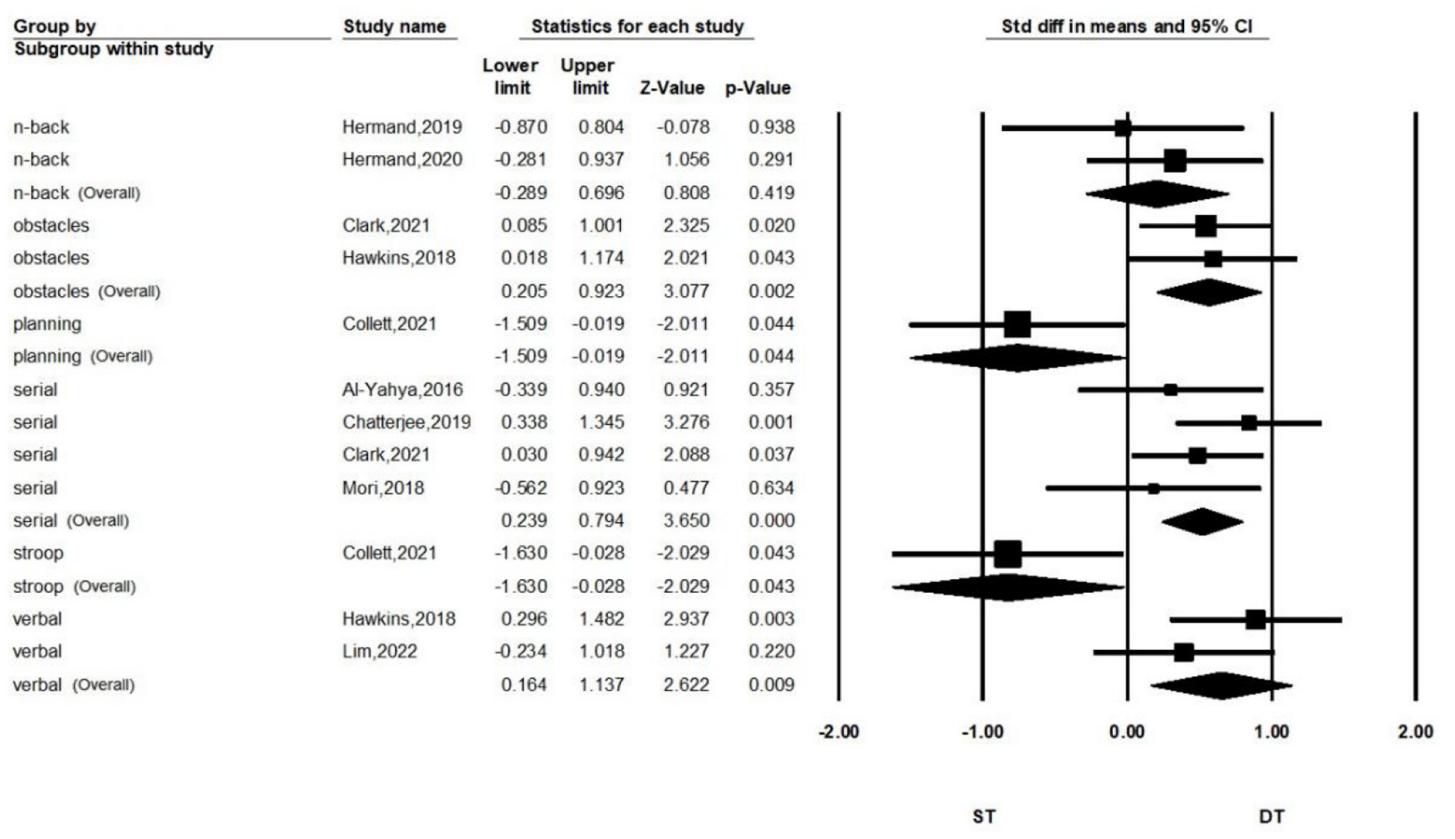
Forest plot for comparing PFC activation of ST and DT in different types of dual task.

Subgroup analyses according to the type of dual-task are shown in Figure 4. We separated the meta-analysis for serial subtraction, obstacle walking, and verbal and n-back tasks. We found that stroke patients who performed the dual-task walking combined with serial subtraction (*SMD* = 0.516, *P* < 0.001, *I*^2^ = 0%, 95% *CI* = 0.239–0.794), obstacle crossing (*SMD* = 0.564, *P* = 0.002, *I*^2^ = 0%, 95% *CI* = 0.205–0.903), or verbal task (*SMD* = 0.654, *P* = 0.009, *I*^2^ = 0%, 95% *CI* = 0.164–1.137) had greater PFC activation than performing single-task walking. Combined with the n-back task, it did not show a significant differentiation of activation compared with single-task walking (*SMD* = 0.203, *P* = 0.419, *I*^2^ = 0%, 95% *CI* = −0.289–0.696).

#### 3.3.2. Sensitivity analyses

**Figure 5:**
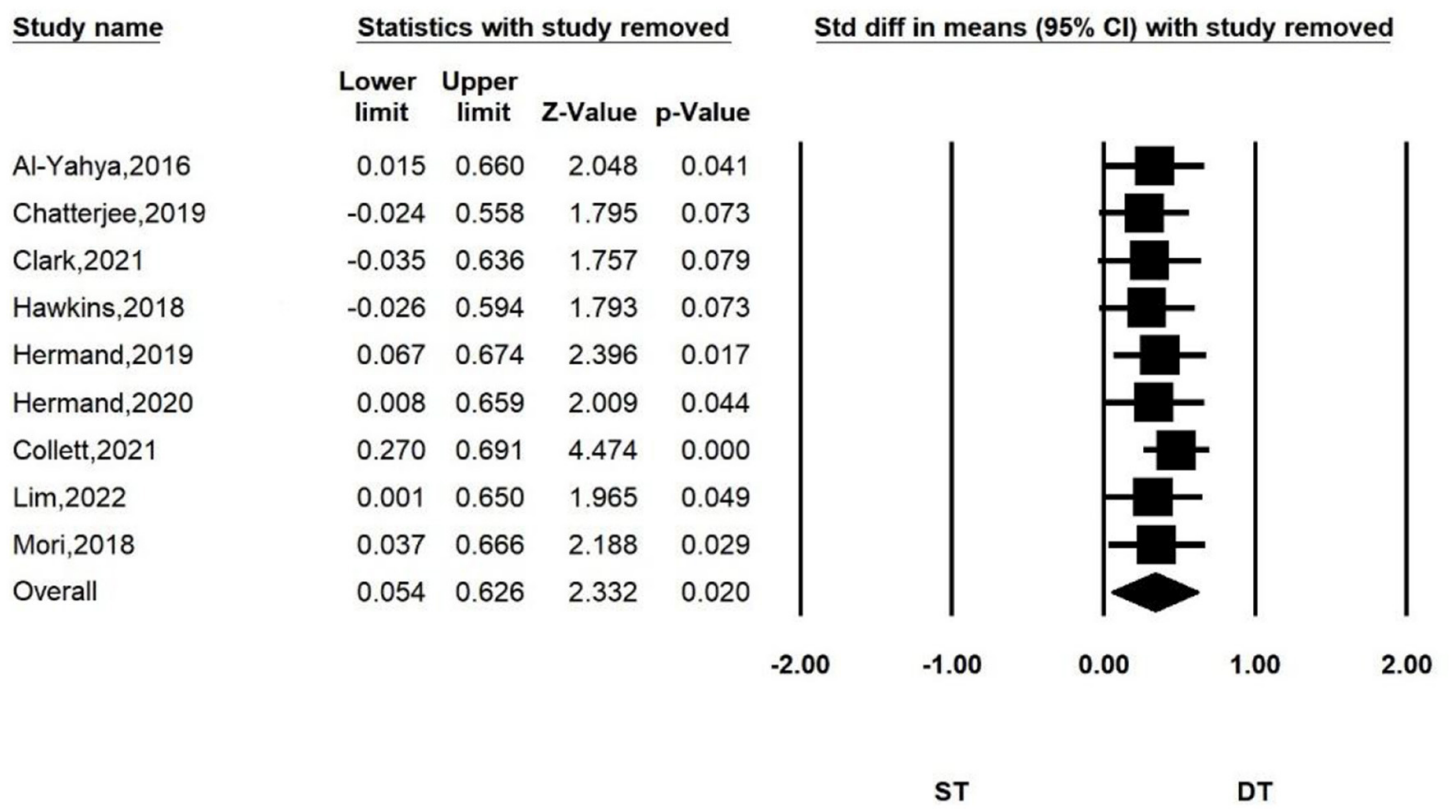
Sensitivity analysis for comparing PFC activation of ST and DT. When the following studies were excluded, the heterogeneity of the combined results was as follows: *I*^2^ = 9.878% (Al-Yahya et al., 2016); *I*^2^ = 6.688% (Chatterjee et al., 2019); *I*^2^ = 3.832% (Clark et al., 2021); *I*^2^ = 5.709% (Hawkins et al., 2018); *I*^2^ = 11.319% (Hermand et al., 2019); *I*^2^ = 9.19% (Hermand et al., 2020); *I*^2^ = 0% (Collett et al., 2021); *I*^2^ = 9.03% (Lim et al., 2022); *I*^2^ = 11.411% (Mori et al., 2018).

To further explore the effect of each article on the total effect size and the accuracy of the results, we performed a sensitivity analysis by sequentially excluding one of the studies, and then repeating the analysis. The results are shown in Figure 5. We found that the significance of the overall effect size changed when some studies were removed. But the results did not change direction. When study by Collett et al. was removed, the heterogeneity index *I*^2^ of the combined results decreased to 0. We considered this study to have a large heterogeneity due to differences in design and equipment. Our results were robust when we included other studies with less heterogeneity.

#### 3.3.3. Publication bias analyses

**Figure 6:**
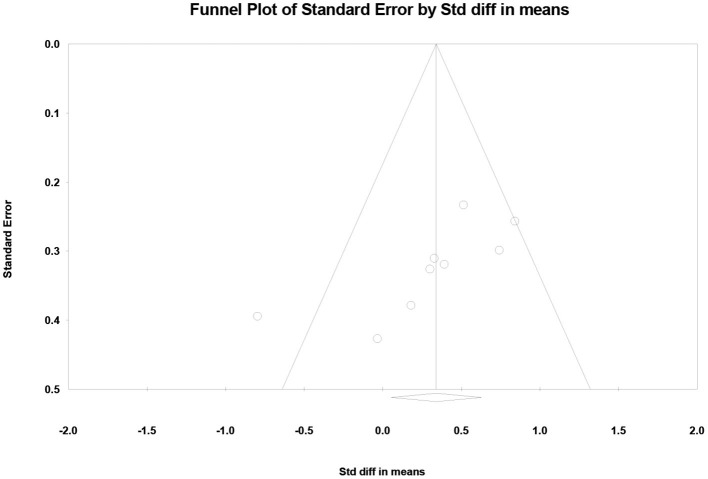
Funnel plots used to examine publication bias in dual task-single task differences in PFC activation.

Figure 6 shows the funnel plot of the nine studies. We performed a publication bias analysis on the standard difference in means between ST and DT. We found that one article fell outside the funnel plot and had a large bias (Collett et al., 2021). The other articles were more evenly distributed and showed no hint of publication bias.

### 3.4. Study quality assessment

Table 3 reports the results of our study quality assessment. All of the eight cross-sectional studies clearly described the research question or purpose, and they followed the inclusion and exclusion criteria of the study. Their outcome measures were also clearly defined and adjusted for the effect of the relationship between exposure and outcome. Five studies examined different levels of the exposure as related to the outcome (Liu et al., 2018; Chatterjee et al., 2019; Hermand et al., 2019, 2020; Lim et al., 2022). Two studies did not implement consistently across all study participants (Hermand et al., 2019, 2020). Only one study provided a clear definition of the study population and reported that the participation rate of eligible persons was at least 50% (Liu et al., 2018). No study provided a sample size justification. Two RCTs used random sequence generation. Participants and assessments were blinded in only one study (Clark et al., 2021). In the other study, only participants were blinded (Collett et al., 2021). All studies included had good study quality. Table 4 shows the quality of the evidence by GRADE criteria. Dual-task had higher PFC activation than single-task, with low certainty of evidence.

**Table 3:** Quality assessment.

**Study name**	**Q1**	**Q2**	**Q3**	**Q4**	**Q5**	**Q6**	**Q7**	**Q8**	**Q9**	**Q10**	**Q11**	**Q12**	**Q13**	**Q14**	**Total score**
**Cross-sectional studies**															
Al-Yahya et al. (2016)	1	0	CD	1	0	0	0	NA	1	NA	1	NA	NA	1	5/10
Hawkins et al. (2018)	1	0	CD	1	0	0	0	NA	1	NA	1	NA	NA	1	5/10
Liu et al. (2018)	1	1	1	1	0	0	0	1	1	NA	1	NA	NA	1	8/11
Mori et al. (2018)	1	0	CD	1	0	0	0	NA	1	NA	1	NA	NA	1	5/10
Chatterjee et al. (2019)	1	0	CD	1	0	0	0	1	1	NA	1	NA	NA	1	6/11
Hermand et al. (2019)	1	0	CD	1	0	0	0	1	0	NA	1	NA	NA	1	5/11
Hermand et al. (2020)	1	0	CD	1	0	0	0	1	0	NA	1	NA	NA	1	5/11
Lim et al. (2022)	1	0	CD	1	0	0	0	1	1	NA	1	NA	NA	1	6/11
**Controlled intervention studies**															
Clark et al. (2021)	1	1	1	1	1	1	1	1	CD	0	1	0	1	1	11/14
Collett et al. (2021)	1	0	1	1	CD	1	1	1	1	1	1	0	1	1	11/14

CD, cannot determine; NA, not applicable.

**Table 4:** The certainty of evidence by GRADE.

**Certainty assessment**							**No.of patients**		**Effect**		**Certainty**
**No.of studies**	**Study design**	**Risk of bias**	**Inconsistency**	**Indirectness**	**Imprecision**	**Other considerations**	**Dual task**	**Single task**	**Relative (95% CI)**	**Absolute (95% CI)**	
10	Observational studies	Not serious^a^	Not serious^b^	Not serious^c^	Serious^d^	All plausible residual confounding would reduce the demonstrated effect^e, f, g, h^	224	224	-	SMD 0.34 (0.054 to 0.626)	⊕⊕○○ Low

CI, confidence interval; SMD, standardized mean difference.

^a^No studies with high risk of bias were identified.

^b^Three studies showed inconsistent results, but the heterogeneity test I^2^ was < 10%.

^c^All studies explored-the association directly.

^d^The sample size of some studies was small and the confidence intervals were wide.

^e^The funnel plot showed that there was no significant publication bias.

^f^The effect sizes were interpreted as 0.2 for a small effect, 0.5 for a moderate effect, and 0.8 or greater for a large effect. This effect size was defined as small.

^g^The two tasks were performed by the same subject to avoid the interference of factors such as the course of the disease, the degree of cognition and the degree of movement.

^h^No study found a dose response gradient.

Study design represents the types of study designs, including randomized trial, observational study and any other evidence. Risk of bias represents the possible deviation between the results of a study and the real situation due to methodological problems. Inconsistency represents whether there are large differences in outcomes between studies.

Indirectness represents whether to compare the efficacy of two interventions directly and indirectly. Imprecision represents effect of the width of the confidence interval on the accuracy of the results. Certainty of evidence reflects the extent to which our confidence in an estimate of the effect is adequate to support a particular recommendation.

⊕⊕⊕⊕ represents “High”; ⊕⊕⊕○ represents “Moderate”; ⊕⊕○○ represents “Low”; ⊕○○○ represents “Very low”.

## 4. Discussion

This study systematically reviews and quantifies the differences in PFC activation between stroke patients performing single-task walking and dual-task walking. Our meta-analysis shows that PFC activation is higher in stroke patients performing dual-task walking than single-task walking. This differences in this PFC activation also vary according to the different types of dual-task and different disease durations in stroke groups.

### 4.1. Activation of PFC: ST vs. DT

Our meta-analysis showed that there was a significant difference in PFC activation between stroke patients performing single-task walking and dual-task walking, which was mainly manifested by an activation degree that was significantly increased during dual-task walking. The following are possible explanations for the results. First, compared with single-task processing, dual-task processing requires additional executive functions to resolve the interference between tasks. Previous studies indicated that the PFC is the main brain region responsible for executive functions in dual-task processing. The PFC has three subregions. The frontopolar cortex is mainly related to coordinating independent tasks processing. The medial frontal cortex forms reward expectations for task setting, based on motivational cues. The lateral prefrontal cortex is mainly responsible for the selection and characterization of task setting rules (Koechlin et al., 2003; Koechlin and Hyafil, 2007). When additional tasks are added, these relevant regions and networks will be mobilized further to complete the dual-task processing. Secondly, this may be the result of the loss of walking automaticity in stroke patients. Healthy individuals can switch intrinsically between automatic and executive control strategies (Clark, 2015). They can reduce the cognitive demands of walking when they perform dual-task walking and use remaining prefrontal resources to complete additional cognitive tasks. The presence of neurological damage in stroke patients exposes dual-task neurocompensatory mechanisms that resulting in a shift in motor strategy from normal automaticity to compensatory. The cognitive control of walking has to be enhanced when automated walking ability is diminished (Ehgoetz Martens et al., 2020). Thirdly, the confidence of patients in their own balance can affect walking performance (Danks et al., 2016). Patients with stroke have deficits in physical functioning, including motor and balance. So they become involuntarily nervous and concentrated when challenged with complex tasks. The fear of falling may massively increase their focus to cope with unfamiliar or unpredictable situations (Aihara et al., 2021). Eventually the patient exhibits a cautious gait and an increase in the control of walking (Rosén et al., 2005; Schinkel-Ivy et al., 2016).

However, a study showed the opposite result. Collett et al. showed that dual task brought lower PFC activity than single task (Collett et al., 2021). We consider that this heterogeneity stems from their different dual-task type. Stroop testing involves the handling of conflicts. Participants are required to perform a less automated task while simultaneously inhibiting a more automated task (Scarpina and Tagini, 2017). The picture planning task involves sequential planning of actions. We presume that the auditory stroop task and picture-planning task in their experiments made it impossible for the subjects to maintain their level of task performance when they reached the upper limits of their available neural resources, and their performances may have declined rapidly (Cabeza et al., 2002). This was similar to the results of an article observing older adults (Salzman et al., 2021). Moreover, one study argued from the concept of resource competition that reduced activation is consistent with the resource allocation model of dual-task processing (Low et al., 2009).

### 4.2. Variations of ST-DT diff: Comparing different disease durations

In our meta-analysis, chronic patients showed small effect sizes, but there were no significant effect sizes in patients in the subacute phase. We interpret that the source of the difference is existence of a “ceiling” phenomenon and can be explained by models of limited capacity. Additional cognitive tasks make it difficult to activate PFC more when the patients already perform single task at or near maximum prefrontal replenishment capacity. This phenomenon has been found in multiple studies (Chatterjee et al., 2019; Hermand et al., 2020). Subacute patients followed the “posture first” strategy to prioritize walking (Ohzuno and Usuda, 2019; Chan and Tsang, 2021). Then, they could only use a small portion of their remaining capacity for additional cognitive tasks, which was limited by a smaller reserve of cognitive resources. A study showed significant cognitive improvement in stroke patients at 3 and 12 months compared to baseline (Buvarp et al., 2021). In the subacute subgroup, the disease duration of the patients in the two included studies was < 3 months, which was in the early subacute phase. At this time, it is still in the stage of motor and cognitive recovery. Another study also found that PFC activation was enhanced in stroke patients after prolonged rehabilitation (Miyai et al., 2003). Therefore, chronic patients may have better cognitive reserves and executive functioning after a period of recovery than those in the subacute phase. They were able to allocate more cognitive resources to cognitive tasks (Hermand et al., 2019). In addition, the subacute patients included in this study mainly underwent the n-back paradigm. This was too difficult to accomplish for subacute patients, who are inherently limited in their abilities. Hermand et al. also found a sharp decrease in the correct response rate when subjects performed the 2-back task, compared to the 1-back task (Hermand et al., 2019). This suggests that patients may drop out of cognitive tasks due to complexity, leading to a not significant ST-DT diff.

### 4.3. Variations of ST-DT diff: Comparing different types of dual-task

Another finding of our study is the different ST-DT diff when stroke patients perform different dual-task types. In our study, serial subtraction, crossing obstacles, and verbal dual tasks all showed moderate effect sizes. The largest effect size of ST-DT diff was found in the verbal task. This is consistent with the results reported by Bishnoi et al. (2021). Verbal tasks involve language-related channels in the frontal lobe. The inferior frontal junction (IFJ) in PFC as the language-related subregion has strong connectivity with known language-related subregions (Hwang et al., 2022). This combined cognitive and linguistic functional demand makes prefrontal activation more pronounced. For the other two dual-task types, a study reported that PFC activation is greater for the cognitive dual-task than for the motor dual-task (Lu et al., 2015). However, our analysis did not show a greater ST-DT diff for the serial subtraction task whilst walking than for the crossing obstacle task. Serial subtraction involves the formation of working memory and concept formation in executive functions while crossing obstacles involves decision planning and the monitoring of actions in executive functions (Darekar et al., 2017; Yang et al., 2018). No matter which dual-task paradigm is used, the brain needs the executive function of the PFC to coordinate and to complete the processing of the two tasks smoothly. Therefore, this caused a similar increase in ST-DT diff.

The n-back task while walking did not show significant ST-DT diff, which is consistent with the results reported by Hermand et al. (2019). This phenomenon may be related to its excessive difficulty. The n-back paradigm involves a mass of working memory. Subjects need to match the stimuli and make specific responses to the information (Li et al., 2021). This is a great challenge for the cognitively impaired stroke patients. They are at increased risk by focusing too much on cognition, so they will prioritize posture control to reduce fall risk (Yogev-Seligmann et al., 2012). At this time, the blood is diverted to other areas that are important for movement, and the transmission of HbO to the PFC is reduced (Beurskens et al., 2014). On the other hand, the inclusion of subacute patients may also have an impact, as mentioned earlier. The results of the more difficult auditory stroop task and the picture-planning task were also explained before.

### 4.4. Clinical implications

It was previously thought that the unique features of the dual-task were allowed to assess the risk of falling and the potential of patients to return to their homes and communities (Hyndman and Ashburn, 2004; Feld et al., 2018; Tsang and Pang, 2020). When patients perform dual task, a poorer performance suggests an increased risk of falls (Wollesen et al., 2019). fNIRS brings more information as another tool. PFC overactivation during dual-task walking often suggests neural inefficiency and is accompanied by a decline in performance ability (Kahya et al., 2019). This indication also signals that the patient is still finding difficulty in walking independently and safely during daily life. Moreover, the variability of ST-DT diff between the dual-task paradigms also implies that different combinations of tasks should be considered during assessment. This will provide a more comprehensive understanding of the deficits in dual-task ability for different stroke patients.

The result may also inform the creation of future intervention programs for the rehabilitation of stroke patients. Many current dual-task trainings aim to use the dual-task practice to improve walking ability and executive function in stroke patients (Liu et al., 2017; Pang et al., 2018; Meester et al., 2019; Strobach, 2020). However, we need to be careful when choosing the cognitive task. We suggest that the intensity of DTI given should increase PFC activation without a significant deterioration in walking performance, in order to achieve the effect of promoting the functional recovery of brain regions and increased dual-task processing capacity. When the activation is too little, one can consider that the cognitive load is too small to achieve the purpose of training, and the other is that the cognitive load is too large, exceeding the upper limit of the patient's ability. This standardized dual-task combination can be used to observe the functional capacity of indicators in more challenging situations than conventional clinical testing, and to individually tailor corresponding interventions for stroke patients.

On the other hand, we do not recommend starting dual-task training in subacute patients, which not only increases the risk during training, but also, the training effect may not meet expectations. One study found that dual-task walking speed increased after dual-task training in patients with better walking abilities, but this was not effective in those with poorer abilities (Collett et al., 2021). We suggest that subacute patients with poorer function should focus more on improving the automation of walking, while chronic patients with better function increase the complexity of cognitive tasks.

### 4.5. Limitations

Our study also had certain limitations. Not only did stroke patients have activity changes in PFC during the performance of dual-task, but other brain regions such as SMA and PMC also underwent characteristic and regular changes in response to increased cognitive tasks. However, these messages were not included in our review, due to the fewer studies analyzing the activations in these brain regions. Additionally, the number of included studies in each of our subgroups varied, which may have biased the review. For example, when we compared the activation differences between subacute and chronic patients, the types of dual-task included in two subgroups included were different. Secondly, we found that most of the included studies used the combined HbO values of the left and right PFC, or compared the difference between the left and right hemispheres. Differences in PFC between the healthy and affected sides were less frequently reported. In general, strokes mostly involve unilateral damage. It is inconclusive as to whether this neural tissue damage makes the results of dual-task response different on the affected side of the brain. Moreover, the number of studies and the limited information available made it difficult to analyze other confounding factors, such as ischemic stroke vs. hemorrhagic stroke, different age groups of the stroke population, and treadmill vs. ground walking. The impacts of these factors should continue to be explored in the future.

## 5. Conclusions

Our meta-analysis shows differences in PFC activation after stroke between the performance of single and dual tasks, based on fNIRS measurements. It varies with the task type and disease duration. The results provide informative suggestions for the future clinical use of the dual-task paradigm to assess the extent of patient walking recovery and training effectiveness, and to predict community walking ability. This also provides a theoretical basis for a better understanding of post-stroke walking behavior deficits and the development of strategies to optimize safe mobility after stroke.

## Data availability statement

The original contributions presented in the study are included in the article, further inquiries can be directed to the corresponding authors.

## Author contributions

QW, SX, WD, and CG chose the topic. QW, SZ, and YSu searched and assessed the studies. WD and SX extracted and analyzed the data. QW, WD, YSh, and CK participated in the writing process. TW, CG, and YZ supervised in the whole process and made the final decision. All authors listed have made a substantial and direct contribution to the work.

## References

[B1] Aihara S. Kitamura S. Dogan M. Sakata S. Kondo K. Otaka Y. (2021). Patients' thoughts on their falls in a rehabilitation hospital: A qualitative study of patients with stroke. BMC Geriatr. 21, 713. 10.1186/s12877-021-02649-1 34922484 PMC8684226

[B2] Al-Yahya E. Johansen-Berg H. Kischka U. Zarei M. Cockburn J. Dawes H. (2016). Prefrontal cortex activation while walking under dual-task conditions in stroke: A multimodal imaging study. Neurorehabil. Neural Repair 30, 591–599. 10.1177/1545968315613864 26493732 PMC5404717

[B3] Baddeley A. Della Sala S. Papagno C. Spinnler H. (1997). Dual-task performance in dysexecutive and nondysexecutive patients with a frontal lesion. Neuropsychology 11, 187–194. 10.1037/0894-4105.11.2.187 9110326

[B4] Bernhardt J. Hayward K. S. Kwakkel G. Ward N. S. Wolf S. L. Borschmann K. . (2017). Agreed definitions and a shared vision for new standards in stroke recovery research: The Stroke Recovery and Rehabilitation Roundtable taskforce. Int. J. Stroke 12, 444–450. 10.1177/1747493017711816 28697708

[B5] Beurskens R. Helmich I. Rein R. Bock O. (2014). Age-related changes in prefrontal activity during walking in dual-task situations: A fNIRS study. Int. J. Psychophysiol. 92, 122–128. 10.1016/j.ijpsycho.2014.03.005 24681355

[B6] Beyaert C. Vasa R. Frykberg G. E. (2015). Gait post-stroke: Pathophysiology and rehabilitation strategies. Neurophysiol. Clin. 45, 335–355. 10.1016/j.neucli.2015.09.005 26547547

[B7] Bishnoi A. Holtzer R. Hernandez M. E. (2021). Brain activation changes while walking in adults with and without neurological disease: Systematic review and meta-analysis of functional near-infrared spectroscopy studies. Brain Sci. 11, 30291. 10.3390/brainsci11030291 33652706 PMC7996848

[B8] Blennerhassett J. M. Levy C. E. Mackintosh A. Yong A. McGinley J. L. (2018). One-quarter of people leave inpatient stroke rehabilitation with physical capacity for community ambulation. J. Stroke Cerebrovasc. Dis. 27, 3404–3410. 10.1016/j.jstrokecerebrovasdis.2018.08.004 30185399

[B9] Buvarp D. Rafsten L. Abzhandadze T. Sunnerhagen K. S. (2021). A prospective cohort study on longitudinal trajectories of cognitive function after stroke. Sci. Rep. 11, 17271. 10.1038/s41598-021-96347-y 34446763 PMC8390476

[B10] Cabeza R. Anderson N. D. Locantore J. K. McIntosh A. R. (2002). Aging gracefully: Compensatory brain activity in high-performing older adults. Neuroimage 17, 1394–1402. 10.1006/nimg.2002.1280 12414279

[B11] Caetano M. J. D. Menant J. C. Schoene D. Pelicioni P. H. S. Sturnieks D. L. Lord S. R. (2017). Sensorimotor and cognitive predictors of impaired gait adaptability in older people. J. Gerontol. A Biol. Sci. Med. Sci. 72, 1257–1263. 10.1093/gerona/glw171 27573810

[B12] Chan W. N. Tsang W. W. N. (2021). Compromised cognition, but not stepping-down performance, when dual-tasking in stroke survivors. J. Mot. Behav. 2021, 1–10. 10.1080/00222895.2021.1918054 34057042

[B13] Chatterjee S. A. Fox E. J. Daly J. J. Rose D. K. Wu S. S. Christou E. A. . (2019). Interpreting prefrontal recruitment during walking after stroke: Influence of individual differences in mobility and cognitive function. Front. Hum. Neurosci. 13, 194. 10.3389/fnhum.2019.00194 31316360 PMC6611435

[B14] Chen W. L. Wagner J. Heugel N. Sugar J. Lee Y. W. Conant L. . (2020). Functional near-infrared spectroscopy and its clinical application in the field of neuroscience: Advances and future directions. Front. Neurosci. 14, 724. 10.3389/fnins.2020.00724 32742257 PMC7364176

[B15] Clark D. J. (2015). Automaticity of walking: Functional significance, mechanisms, measurement and rehabilitation strategies. Front. Hum. Neurosci. 9, 246. 10.3389/fnhum.2015.00246 25999838 PMC4419715

[B16] Clark D. J. Rose D. K. Butera K. A. Hoisington B. DeMark L. Chatterjee S. A. . (2021). Rehabilitation with accurate adaptability walking tasks or steady state walking: A randomized clinical trial in adults post-stroke. Clin. Rehabil. 35, 1196–1206. 10.1177/02692155211001682 33722075 PMC10416755

[B17] Collett J. Fleming M. K. Meester D. Al-Yahya E. Wade D. T. Dennis A. . (2021). Dual-task walking and automaticity after stroke: Insights from a secondary analysis and imaging sub-study of a randomised controlled trial. Clin. Rehabil. 35, 1599–1610. 10.1177/02692155211017360 34053250 PMC8524683

[B18] Collette F. Olivier L. Van der Linden M. Laureys S. Delfiore G. Luxen A. . (2005). Involvement of both prefrontal and inferior parietal cortex in dual-task performance. Brain Res. Cogn. Brain Res. 24, 237–251. 10.1016/j.cogbrainres.2005.01.023 15993762

[B19] Danks K. A. Pohlig R. T. Roos M. Wright T. R. Reisman D. S. (2016). Relationship between walking capacity, biopsychosocial factors, self-efficacy, and walking activity in persons poststroke. J. Neurol Phys. Ther. 40, 232–238. 10.1097/NPT.0000000000000143 27548750 PMC5025374

[B20] Darekar A. Lamontagne A. Fung J. (2017). Locomotor circumvention strategies are altered by stroke: I. Obstacle clearance. J. Neuroeng. Rehabil. 14, 56. 10.1186/s12984-017-0264-8 28615042 PMC5471680

[B21] Delavaran H. Jönsson A. C. Lövkvist H. Iwarsson S. Elmståhl S. Norrving B. . (2017). Cognitive function in stroke survivors: A 10-year follow-up study. Acta Neurol. Scand. 136, 187–194. 10.1111/ane.12709 27804110

[B22] Ehgoetz Martens K. A. Matar E. Shine J. M. Phillips J. R. Georgiades M. J. Grunstein R. R. . (2020). The neural signature of impaired dual-tasking in idiopathic rapid eye movement sleep behavior disorder patients. Mov. Disord. 35, 1596–1606. 10.1002/mds.28114 32525224

[B23] Feld J. A. Zukowski L. A. Howard A. G. Giuliani C. A. Altmann L. J. P. Najafi B. . (2018). Relationship between dual-task gait speed and walking activity poststroke. Stroke 49, 1296–1298. 10.1161/STROKEAHA.117.019694 29622624 PMC6034633

[B24] Gramigna V. Pellegrino G. Cerasa A. Cutini S. Vasta R. Olivadese G. . (2017). Near-infrared spectroscopy in gait disorders: Is it time to begin? Neurorehabil. Neural Repair 31, 402–412. 10.1177/1545968317693304 28196453

[B25] Hamacher D. Herold F. Wiegel P. Hamacher D. Schega L. (2015). Brain activity during walking: A systematic review. Neurosci. Biobehav. Rev. 57, 310–327. 10.1016/j.neubiorev.2015.08.002 26306029

[B26] Handy T. C. (2000). Capacity theory as a model of cortical behavior. J. Cogn. Neurosci. 12, 1066–1069. 10.1162/08989290051137576 11177425

[B27] Hawkins K. A. Fox E. J. Daly J. J. Rose D. K. Christou E. A. McGuirk T. E. . (2018). Prefrontal over-activation during walking in people with mobility deficits: Interpretation and functional implications. Hum. Mov. Sci. 59, 46–55. 10.1016/j.humov.2018.03.010 29604488 PMC5988641

[B28] Hermand E. Compagnat M. Dupuy O. Salle J. Y. Daviet J. C. Perrochon A. (2020). Functional status is associated with prefrontal cortex activation in gait in subacute stroke patients: A functional near-infrared spectroscopy study. Front. Neurol. 11, 559227. 10.3389/fneur.2020.559227 33224085 PMC7674599

[B29] Hermand E. Tapie B. Dupuy O. Fraser S. Compagnat M. Salle J. Y. . (2019). Prefrontal cortex activation during dual task with increasing cognitive load in subacute stroke patients: A pilot study. Front. Aging Neurosci. 11, 160. 10.3389/fnagi.2019.00160 31312136 PMC6614381

[B30] Hoshi Y. Kobayashi N. Tamura M. (2001). Interpretation of near-infrared spectroscopy signals: A study with a newly developed perfused rat brain model. J. Appl. Physiol. 90, 1657–1662. 10.1152/jappl.2001.90.5.1657 11299252

[B31] Hwang Y. E. Kim Y. B. Son Y. D. (2022). Finding cortical subregions regarding the dorsal language pathway based on the structural connectivity. Front. Hum. Neurosci. 16, 784340. 10.3389/fnhum.2022.784340 35585994 PMC9108242

[B32] Hyndman D. Ashburn A. (2004). Stops walking when talking as a predictor of falls in people with stroke living in the community. J. Neurol Neurosurg. Psychiatry 75, 994–997. 10.1136/jnnp.2003.016014 15201358 PMC1739145

[B33] Iqbal M. Arsh A. Hammad S. M. Haq I. U. Darain H. (2020). Comparison of dual task specific training and conventional physical therapy in ambulation of hemiplegic stroke patients: A randomized controlled trial. J. Pak. Med. Assoc. 70, 7–10. 10.5455/J.P.M.A.10443 31954014

[B34] Jasdzewski G. Strangman G. Wagner J. Kwong K. K. Poldrack R. A. Boas D. A. (2003). Differences in the hemodynamic response to event-related motor and visual paradigms as measured by near-infrared spectroscopy. Neuroimage 20, 479–488. 10.1016/S1053-8119(03)00311-2 14527608

[B35] Kahya M. Moon S. Ranchet M. Vukas R. R. Lyons K. E. Pahwa R. . (2019). Brain activity during dual task gait and balance in aging and age-related neurodegenerative conditions: A systematic review. Exp. Gerontol. 128, 110756. 10.1016/j.exger.2019.110756 31648005 PMC6876748

[B36] Kim H. Y. Seo K. Jeon H. J. Lee U. Lee H. (2017). Application of functional near-infrared spectroscopy to the study of brain function in humans and animal models. Mol. Cells 40, 523–532. 10.14348/molcells.2017.0153 28835022 PMC5582298

[B37] Koechlin E. Hyafil A. (2007). Anterior prefrontal function and the limits of human decision-making. Science 318, 594–598. 10.1126/science.1142995 17962551

[B38] Koechlin E. Ody C. Kouneiher F. (2003). The architecture of cognitive control in the human prefrontal cortex. Science 302, 1181–1185. 10.1126/science.1088545 14615530

[B39] Kondo H. Osaka N. Osaka M. (2004). Cooperation of the anterior cingulate cortex and dorsolateral prefrontal cortex for attention shifting. Neuroimage 23, 670–679. 10.1016/j.neuroimage.2004.06.014 15488417

[B40] Leff D. R. Orihuela-Espina F. Elwell C. E. Athanasiou T. Delpy D. T. Darzi A. W. . (2011). Assessment of the cerebral cortex during motor task behaviours in adults: A systematic review of functional near infrared spectroscopy (fNIRS) studies. Neuroimage 54, 2922–2936. 10.1016/j.neuroimage.2010.10.058 21029781

[B41] Li W. Zhang Q. Qiao H. Jin D. Ngetich R. K. Zhang J. . (2021). Dual n-back working memory training evinces superior transfer effects compared to the method of loci. Sci. Rep. 11, 3072. 10.1038/s41598-021-82663-w 33542383 PMC7862396

[B42] Lim S. B. Louie D. R. Peters S. Liu-Ambrose T. Boyd L. A. Eng J. J. (2021). Brain activity during real-time walking and with walking interventions after stroke: A systematic review. J. Neuroeng. Rehabil. 18, 8. 10.1186/s12984-020-00797-w 33451346 PMC7811232

[B43] Lim S. B. Peters S. Yang C. L. Boyd L. A. Liu-Ambrose T. Eng J. J. (2022). Frontal, sensorimotor, and posterior parietal regions are involved in dual-task walking after stroke. Front. Neurol. 13, 904145. 10.3389/fneur.2022.904145 35812105 PMC9256933

[B44] Liu Y. C. Yang Y. R. Tsai Y. A. Wang R. Y. (2017). Cognitive and motor dual task gait training improve dual task gait performance after stroke—A randomized controlled pilot trial. Sci. Rep. 7, 4070. 10.1038/s41598-017-04165-y 28642466 PMC5481328

[B45] Liu Y. C. Yang Y. R. Tsai Y. A. Wang R. Y. Lu C. F. (2018). Brain activation and gait alteration during cognitive and motor dual task walking in stroke—A functional near-infrared spectroscopy study. IEEE Trans. Neural Syst. Rehabil. Eng. 26, 2416–2423. 10.1109/TNSRE.2018.2878045 30371378

[B46] Lord S. E. McPherson K. McNaughton H. K. Rochester L. Weatherall M. (2004). Community ambulation after stroke: How important and obtainable is it and what measures appear predictive? Arch. Phys. Med. Rehabil. 85, 234–239. 10.1016/j.apmr.2003.05.002 14966707

[B47] Low K. A. Leaver E. E. Kramer A. F. Fabiani M. Gratton G. (2009). Share or compete? Load-dependent recruitment of prefrontal cortex during dual-task performance. Psychophysiology 46, 1069–1079. 10.1111/j.1469-8986.2009.00854.x 19572909 PMC2746863

[B48] Lu C. F. Liu Y. C. Yang Y. R. Wu Y. T. Wang R. Y. (2015). Maintaining gait performance by cortical activation during dual-task interference: A functional near-infrared spectroscopy study. PLoS ONE 10, e0129390. 10.1145/2818302 26079605 PMC4469417

[B49] Meester D. Al-Yahya E. Dennis A. Collett J. Wade D. T. Ovington M. . (2019). A randomized controlled trial of a walking training with simultaneous cognitive demand (dual-task) in chronic stroke. Eur. J. Neurol. 26, 435–441. 10.1111/ene.13833 30308699 PMC6824903

[B50] Mehagnoul-Schipper D. J. van der Kallen B. F. Colier W. N. van der Sluijs M. C. van Erning L. J. Thijssen H. O. . (2002). Simultaneous measurements of cerebral oxygenation changes during brain activation by near-infrared spectroscopy and functional magnetic resonance imaging in healthy young and elderly subjects. Hum. Brain Mapp. 16, 14–23. 10.1002/hbm.10026 11870923 PMC6871837

[B51] Min B. K. Kim H. S. Ko W. Ahn M. H. Suk H. I. Pantazis D. . (2021). Electrophysiological decoding of spatial and color processing in human prefrontal cortex. Neuroimage 237, 118165. 10.1016/j.neuroimage.2021.118165 34000400 PMC8344402

[B52] Miyai I. Yagura H. Hatakenaka M. Oda I. Konishi I. Kubota K. (2003). Longitudinal optical imaging study for locomotor recovery after stroke. Stroke 34, 2866–2870. 10.1161/01.STR.0000100166.81077.8A 14615624

[B53] Moher D. Liberati A. Tetzlaff J. Altman D. G. (2009). Preferred reporting items for systematic reviews and meta-analyses: The PRISMA statement. Br. Med. J. 339, b2535. 10.1136/bmj.b2535 19622551 PMC2714657

[B54] Moon Y. Sung J. An R. Hernandez M. E. Sosnoff J. J. (2016). Gait variability in people with neurological disorders: A systematic review and meta-analysis. Hum. Mov. Sci. 47, 197–208. 10.1016/j.humov.2016.03.010 27023045

[B55] Mori T. Takeuchi N. Izumi S. I. (2018). Prefrontal cortex activation during a dual task in patients with stroke. Gait Posture 59, 193–198. 10.1016/j.gaitpost.2017.09.032 29073516

[B56] Ohzuno T. Usuda S. (2019). Cognitive-motor interference in post-stroke individuals and healthy adults under different cognitive load and task prioritization conditions. J. Phys. Ther. Sci. 31, 255–260. 10.1589/jpts.31.255 30936641 PMC6428651

[B57] Pang M. Y. C. Yang L. Ouyang H. Lam F. M. H. Huang M. Jehu D. A. (2018). Dual-task exercise reduces cognitive-motor interference in walking and falls after stroke. Stroke 49, 2990–2998. 10.1161/STROKEAHA.118.022157 30571419

[B58] Parris B. A. Wadsley M. G. Hasshim N. Benattayallah A. Augustinova M. Ferrand L. (2019). An fMRI study of response and semantic conflict in the stroop task. Front. Psychol. 10, 2426. 10.3389/fpsyg.2019.02426 31736827 PMC6834775

[B59] Pashler H. (1994). Dual-task interference in simple tasks: Data and theory. Psychol. Bull. 116, 220–244. 10.1037/0033-2909.116.2.220 7972591

[B60] Pei L. Wu H. (2019). Does online learning work better than offline learning in undergraduate medical education? A systematic review and meta-analysis. Med. Educ. Onl. 24, 1666538. 10.1080/10872981.2019.1666538 31526248 PMC6758693

[B61] Perrey S. (2014). Possibilities for examining the neural control of gait in humans with fNIRS. Front. Physiol. 5, 204. 10.3389/fphys.2014.00204 24904433 PMC4035560

[B62] Pinti P. Tachtsidis I. Hamilton A. Hirsch J. Aichelburg C. Gilbert S. . (2020). The present and future use of functional near-infrared spectroscopy (fNIRS) for cognitive neuroscience. Ann. N. Y. Acad. Sci. 1464, 5–29. 10.1111/nyas.13948 30085354 PMC6367070

[B63] Rosén E. Sunnerhagen K. S. Kreuter M. (2005). Fear of falling, balance, and gait velocity in patients with stroke. Physiother. Theory Pract. 21, 113–120. 10.1080/09593980590922299 16392464

[B64] Saini V. Guada L. Yavagal D. R. (2021). Global epidemiology of stroke and access to acute ischemic stroke interventions. Neurology 97(20 Suppl.2), S6–s16. 10.1212/WNL.0000000000012781 34785599

[B65] Salzman T. Tobón Vallejo D. Polskaia N. Michaud L. St-Amant G. Lajoie Y. . (2021). Hemodynamic and behavioral changes in older adults during cognitively demanding dual tasks. Brain Behav. 11, e02021. 10.1002/brb3.2021 33417301 PMC7994703

[B66] Scarpina F. Tagini S. (2017). The stroop color and word test. Front. Psychol. 8, 557. 10.3389/fpsyg.2017.00557 28446889 PMC5388755

[B67] Schinkel-Ivy A. Inness E. L. Mansfield A. (2016). Relationships Between Fear of Falling, Balance Confidence, and Control of Balance, Gait, and Reactive Stepping in Individuals With Sub-acute Stroke. [1879-2219 (Electronic)]. 26482234 10.1016/j.gaitpost.2015.09.015 PMC5045898

[B68] Strobach T. (2020). The dual-task practice advantage: Empirical evidence and cognitive mechanisms. Psychon. Bull. Rev. 27, 3–14. 10.3758/s13423-019-01619-4 31152433

[B69] Tsang C. S. Wang S. Miller T. Pang M. Y. (2022). Degree and pattern of dual-task interference during walking vary with component tasks in people after stroke: A systematic review. J. Physiother. 68, 26–36. 10.1016/j.jphys.2021.12.009 34953757

[B70] Tsang C. S. L. Pang M. Y. C. (2020). Association of subsequent falls with evidence of dual-task interference while walking in community-dwelling individuals after stroke. Clin. Rehabil. 34, 971–980. 10.1177/0269215520923700 32460556

[B71] Udina C. Avtzi S. Durduran T. Holtzer R. Rosso A. L. Castellano-Tejedor C. . (2019). Functional near-infrared spectroscopy to study cerebral hemodynamics in older adults during cognitive and motor tasks: A review. Front. Aging Neurosci. 11, 367. 10.3389/fnagi.2019.00367 32038224 PMC6985209

[B72] Veldkamp R. Goetschalckx M. Hulst H. E. Nieuwboer A. Grieten K. Baert I. . (2021). Cognitive-motor interference in individuals with a neurologic disorder: A systematic review of neural correlates. Cogn. Behav. Neurol. 34, 79–95. 10.1097/WNN.0000000000000269 34074863

[B73] Wickens C. D. (2002). Multiple resources and performance prediction. Theoret. Iss. Erg. Sci. 3, 159–177. 10.1080/14639220210123806

[B74] Wollesen B. Wanstrath M. van Schooten K. S. Delbaere K. (2019). A taxonomy of cognitive tasks to evaluate cognitive-motor interference on spatiotemoporal gait parameters in older people: A systematic review and meta-analysis. Eur. Rev. Aging Phys. Act. 16, 12. 10.1186/s11556-019-0218-1 31372186 PMC6661106

[B75] Yang L. Lam F. M. Huang M. He C. Pang M. Y. (2018). Dual-task mobility among individuals with chronic stroke: Changes in cognitive-motor interference patterns and relationship to difficulty level of mobility and cognitive tasks. Eur. J. Phys. Rehabil. Med. 54, 526–535. 10.23736/S1973-9087.17.04773-6 28949119

[B76] Yogev-Seligmann G. Hausdorff J. M. Giladi N. (2012). Do we always prioritize balance when walking? Towards an integrated model of task prioritization. Mov. Disord. 27, 765–770. 10.1002/mds.24963 22419512

[B77] Young M. J. Regenhardt R. W. Leslie-Mazwi T. M. Stein M. A. (2020). Disabling stroke in persons already with a disability: Ethical dimensions and directives. Neurology 94, 306–310. 10.1212/WNL.0000000000008964 31969466 PMC7176295

