# Supplementary material for: Brain activation of the PFC during dual-task walking in stroke patients: A systematic review and meta-analysis of functional near-infrared spectroscopy studies
Source: Front Neurosci. 2023 Feb 16;17:1111274. doi: 10.3389/fnins.2023.1111274 (PMC9980909; doi:10.3389/fnins.2023.1111274)
Supplement: Supplementary file 1 [file Table_1.DOCX]

**Supplementary Material**

| **Database** | **Search algorithm** |
| --- | --- |
| Medline | (((TS=(stroke)) AND TS=((dual task) OR (walking) OR (gait) OR (locomotion) OR (mobility) OR (ambulation) OR (lower limb movement) OR (lower limb motor)))) AND TS=((fNIRS) OR (functional near infrared spectroscopy) OR (Functional Near-Infrared spectroscopy) OR (NIRS) OR (near-infrared spectroscopy)) |
| Embase | ('stroke'/exp OR stroke) AND ('dual task'/exp OR 'dual task' OR (dual AND ('task'/exp OR task)) OR 'walking'/exp OR walking OR 'gait'/exp OR gait OR 'locomotion'/exp OR locomotion OR 'mobility'/exp OR mobility OR 'ambulation'/exp OR ambulation OR 'lower limb movement' OR (lower AND ('limb'/exp OR limb) AND ('movement'/exp OR movement)) OR 'lower limb motor' OR (lower AND ('limb'/exp OR limb) AND ('motor'/exp OR motor))) AND ('fnirs'/exp OR fnirs OR 'functional near infrared spectroscopy'/exp OR 'functional near infrared spectroscopy' OR (functional AND near AND ('infrared'/exp OR infrared) AND ('spectroscopy'/exp OR spectroscopy)) OR 'functional near-infrared spectroscopy'/exp OR 'functional near-infrared spectroscopy' OR (functional AND ('near infrared'/exp OR 'near infrared') AND ('spectroscopy'/exp OR spectroscopy)) OR 'nirs'/exp OR nirs OR 'near-infrared spectroscopy'/exp OR 'near-infrared spectroscopy' OR (('near infrared'/exp OR 'near infrared') AND ('spectroscopy'/exp OR spectroscopy)) OR 'near infrared spectroscopy'/exp OR 'near infrared spectroscopy' OR (near AND ('infrared'/exp OR infrared) AND ('spectroscopy'/exp OR spectroscopy))) |
| PubMed | ((stroke) AND ((dual task) OR (walking) OR (gait) OR (locomotion) OR (mobility) OR (ambulation) OR (lower limb movement) OR (lower limb motor))) AND ((fNIRS) OR (functional near infrared spectroscopy) OR (Functional Near-Infrared spectroscopy) OR (NIRS) OR (near-infrared spectroscopy) OR (near infrared spectroscopy)) |
| Web of Science | (((TS=(stroke)) AND TS=((dual task) OR (walking) OR (gait) OR (locomotion) OR (mobility) OR (ambulation) OR (lower limb movement) OR (lower limb motor)))) AND TS=((fNIRS) OR (functional near infrared spectroscopy) OR (Functional Near-Infrared spectroscopy) OR (NIRS) OR (near-infrared spectroscopy)) |
| CINAHL | ((stroke) AND ((dual task) OR (walking) OR (gait) OR (locomotion) OR (mobility) OR (ambulation) OR (lower limb movement) OR (lower limb motor))) AND ((fNIRS) OR (functional near infrared spectroscopy) OR (Functional Near-Infrared spectroscopy) OR (NIRS) OR (near-infrared spectroscopy) OR (near infrared spectroscopy)) |
| Cochrane Library | "stroke" and ("dual task" or walking or gait or locomotion or mobility or ambulation or "lower limb movement" or "lower limb motor") and ("fNIRS" or "Functional Near‐infrared spectroscopy" or "Functional Near infrared spectroscopy" or "NIRS" or "Near‐infrared spectroscopy") |
